# Supplementary material for: Elements of a stochastic 3D prediction engine in larval zebrafish prey capture
Source: eLife. 2019 Nov 26;8:e51975. doi: 10.7554/eLife.51975 (PMC6930116; doi:10.7554/eLife.51975)
Supplement: Figure 2—source data 1. [file elife-51975-fig2-data1.zip › Figure 2 Source/Notes on Source Data.docx]

Notes on Source Data:

1. All_huntbouts_source.csv composes figures 2, 3, and 5 as well as their figure supplements. It is also used for generating the real fish data in Figure 6A. Notes on its contents are below in points 2-6.
2. Strike Or Abort Header indicates whether bout occurred during a sequence leading to a strike (1 or 2), an abort (3), or a change of mind (4+).
3. Bout Number 0 is the initiation bout, negative numbers are the final bouts of the sequence (aborts or strikes), and all positive numbers are pursuit bouts.
4. Azimuth and altitude coordinates in the source data are in radians and must be converted to degrees to obtain plots in main paper.
5. Distance in source file is in pixels, which are .0106 mm.
6. Bout Durations are in seconds.
7. Figure 1 – figure supplement 2 is generated from all_stimuli_source.csv and all_stimuli_random_source.csv.
8. Bdb_marr.bdb is a BayesDB file containing the generators for DPMMs used in Figure 6. BayesDB, which runs in a Jupyter notebook interface supplied by a Docker image, can be obtained open source from the MIT Probabilistic Computing Project lead by VKM.
