# Supplementary material for: Elements of a stochastic 3D prediction engine in larval zebrafish prey capture
Source: eLife. 2019 Nov 26;8:e51975. doi: 10.7554/eLife.51975 (PMC6930116; doi:10.7554/eLife.51975)
Supplement: Transparent reporting form [file elife-51975-transrepform.docx]

***eLife’s* transparent reporting form**

We encourage authors to provide detailed information *within their submission* to facilitate the interpretation and replication of experiments. Authors can upload supporting documentation to indicate the use of appropriate reporting guidelines for health-related research (see [EQUATOR Network](http://www.equator-network.org/%20)), life science research (see the [BioSharing Information Resource](https://biosharing.org/)), or the [ARRIVE guidelines](http://www.plosbiology.org/article/info:doi/10.1371/journal.pbio.1000412) for reporting work involving animal research. Where applicable, authors should refer to any relevant reporting standards documents in this form.

If you have any questions, please consult our Journal Policies and/or contact us: [editorial@elifesciences.org](mailto:editorial@elifesciences.org).

**Sample-size estimation**

- You should state whether an appropriate sample size was computed when the study was being designed
- You should state the statistical method of sample size computation and any required assumptions
- If no explicit power analysis was used, you should describe how you decided what sample (replicate) size (number) to use

Please outline where this information can be found within the submission (e.g., sections or figure legends), or explain why this information doesn’t apply to your submission:

Since we did not set out to obtain any particular “result” (i.e. we only wanted to characterize the fish’s strategy regardless of outcome), we simply designed the study so that we would obtain ~1000 bouts worth of data during successful hunting sequences. We reasoned that 50 fish striking at ~5 prey per experiment should yield this threshold, which it did.

**Replicates**

- You should report how often each experiment was performed
- You should include a definition of biological versus technical replication
- The data obtained should be provided and sufficient information should be provided to indicate the number of independent biological and/or technical replicates
- If you encountered any outliers, you should describe how these were handled
- Criteria for exclusion/inclusion of data should be clearly stated
- High-throughput sequence data should be uploaded before submission, with a private link for reviewers provided (these are available from both GEO and ArrayExpress)

Please outline where this information can be found within the submission (e.g., sections or figure legends), or explain why this information doesn’t apply to your submission:

Technical replication is not applicable to this paper; each experiment is a fish, which is a biological replicate. 7 out of 53 fish were discarded for not capturing more than 1 prey per 10 minute experiment as a quality control for larvae health, which is described in Methods. There was only a very small set of outliers (12 points out of 1784) that weren’t used for regression fitting in Figure 5A; this is due to the lack of relationship between the fish’s bout distance and prey distance when prey are further than 6 mm, which is explained in the 5A legend.

**Statistical reporting**

- Statistical analysis methods should be described and justified
- Raw data should be presented in figures whenever informative to do so (typically when N per group is less than 10)
- For each experiment, you should identify the statistical tests used, exact values of N, definitions of center, methods of multiple test correction, and dispersion and precision measures (e.g., mean, median, SD, SEM, confidence intervals; and, for the major substantive results, a measure of effect size (e.g., Pearson's r, Cohen's d)
- Report exact p-values wherever possible alongside the summary statistics and 95% confidence intervals. These should be reported for all key questions and not only when the p-value is less than 0.05.

Please outline where this information can be found within the submission (e.g., sections or figure legends), or explain why this information doesn’t apply to your submission:

Justification of statistical methods and analysis choices are defined in text and legends; 95% CIs and test results are reported where fits and tests were performed, both graphically, in text, and in legends. Pearsons r’s are included for each regression fit in the figures.

(For large datasets, or papers with a very large number of statistical tests, you may upload a single table file with tests, Ns, etc., with reference to sections in the manuscript.)

**Group allocation**

- Indicate how samples were allocated into experimental groups (in the case of clinical studies, please specify allocation to treatment method); if randomization was used, please also state if restricted randomization was applied
- Indicate if masking was used during group allocation, data collection and/or data analysis

Please outline where this information can be found within the submission (e.g., sections or figure legends), or explain why this information doesn’t apply to your submission:

N/A.

**Additional data files (“source data”)**

- We encourage you to upload relevant additional data files, such as numerical data that are represented as a graph in a figure, or as a summary table
- Where provided, these should be in the most useful format, and they can be uploaded as “Source data” files linked to a main figure or table
- Include model definition files including the full list of parameters used
- Include code used for data analysis (e.g., R, MatLab)
- Avoid stating that data files are “available upon request”

Please indicate the figures or tables for which source data files have been provided:

All software related to behavioral analysis, modeling, and virtual prey capture simulation is freely available at [www.github.com/larrylegend33/PreycapMaster](http://www.github.com/larrylegend33/PreycapMaster). The software is licensed under a GNU General Public License 3.0. Source data for analysis and simulations is enclosed as “Source Data” in relevant figures. Source Data for describing the statistics of chosen prey are included in Figure 1 – figure supplement 2. Source Data for Figure 2 contains all pursuit bouts analyzed in the dataset; it was used to construct Figures 2, 3, 5, and 6A, and is accompanied by instructions for running queries. Source Data for Figure 6 contains the generators for simulating from the DPMMs in Figure 6. Using the code at [www.github.com/larrylegend33/PreycapMaster](http://www.github.com/larrylegend33/PreycapMaster) and the generators in Source Data – Figure 6 requires obtaining the BayesDB software package, which is freely available at <http://probcomp.csail.mit.edu/software/bayesdb/>.
